# Tracing curves in the plane: Geometric-invariant learning from human demonstrations

**DOI:** 10.1371/journal.pone.0294046

**Published:** 2024-02-28

**Authors:** Sri Harsha Turlapati, Lyudmila Grigoryeva, Juan-Pablo Ortega, Domenico Campolo

**Affiliations:** 1 School of Mechanical and Aerospace Engineering, Nanyang Technological University, Singapore, Singapore; 2 Faculty of Mathematics and Statistics, University of St. Gallen, St. Gallen, Switzerland; 3 Department of Statistics, University of Warwick, Coventry, United Kingdom; 4 School of Physical and Mathematical Sciences, Nanyang Technological University, Singapore, Singapore; Sun Yat-sen University, CHINA

## Abstract

The empirical laws governing human-curvilinear movements have been studied using various relationships, including minimum jerk, the 2/3 power law, and the piecewise power law. These laws quantify the speed-curvature relationships of human movements during curve tracing using critical speed and curvature as regressors. In this work, we provide a reservoir computing-based framework that can learn and reproduce human-like movements. Specifically, the geometric invariance of the observations, i.e., lateral distance from the closest point on the curve, instantaneous velocity, and curvature, when viewed from the moving frame of reference, are exploited to train the reservoir system. The artificially produced movements are evaluated using the power law to assess whether they are indistinguishable from their human counterparts. The generalisation capabilities of the trained reservoir to curves that have not been used during training are also shown.

## 1 Introduction

For centuries, human motion has been the focus of studies by scientists and artists. Only in the 19th century, quantitative methods started to emerge [1] which allowed, among other things, formulating *empirical laws* to capture the apparent regularities behind our seemingly effortless motion. As highlighted by Ajemian and Hogan [2], just like what happened in astronomy, progress in motor sciences can be divided into three stages: from “heuristic description”, to “curve fitting”, and to “theoretical synthesis”.

Modern theoretical neuroscience is less than a century old while robotics, another discipline interested in humans, is much younger (in its infancy, one would say, judging from the gap between robots and the simplest forms of animal intelligence). One area where robotics has much to learn from neuroscience is *movement synthesis*.

Perhaps the only genuine similarity between humans and robots of similar size (we are not concerned here with milli-, micro-, or nano-robots) is the fact they both operate in the physical world and are subject to the laws of Mechanics. These laws are very often expressed in geometric forms, e.g. where deviations from “straightness” (e.g. curvature) are related to external forces. These laws are to be obeyed by both humans and robots. Unlike an apple falling from a tree, which can just follow Newton’s laws, humans (and biological agents, in general) can exert various forms of control, albeit still constrained by the very same laws affecting the apple. For example, this translates into an excess of ‘degrees of freedom’, both kinematic and muscular, which the brain is free to choose from, leading to the concept of *synergies*, a classical problem too vast to review here (the reader is referred to textbooks such as [3, 4]).

While the works above mainly focus on the problem of mapping a redundant kinematic or muscular space onto a task space, typically with much fewer degrees of freedom, the problem addressed in this work can be defined on the task space itself (where the planning is thought to mainly take place [5]) and relates to *curve-tracing tasks*. Since the 80s, studies on curve tracing have focused on both cognitive [6] as well as on motor aspects [7]. In the framework of planar hand movements, where subjects are instructed to freely draw 2D curves, a remarkable regularity has been observed, at any given time, between the velocity *v* along the curve and the curvature itself *κ* which follows the relation:

$$v\propto \kappa^{\beta }\;\Leftrightarrow \;\text{log}\;v=\beta \;\text{log}\;\kappa +const,$$

where *β* = −1/3.

For historical reasons, this was named the “2/3 power law” [8] and, in the last four decades, it has been tested, applied and extended under the general name of power laws [9] (the reader is referred to [10] for a recent review). Beyond curve tracing, the validity of the 2/3 power law has been established for a variety of human tasks including hand movement [11], eye movement [12] and locomotion [13, 14]. Apart from the kinematic relationship, the power law has also been investigated in dynamic constraints [15]. This speed curvature relation with nearly constant speed along a constant curvature paths was also observed in mechanically constrained tasks [16]. Power laws, however, are of empirical nature and only hold in a statistical sense. To account for deviations from the ‘pure’ 2/3 value, several extensions to the power law have been proposed, which include additional parameters to better fit data. Bennequin et al. [9], proposed a piecewise linear relationship characterized by a ‘critical’ curvature *κ*_*c*_ and velocity *v*_*c*_, to be better detailed later. Such additional parameters, once regressed, carry subject-specific information which could be used for the generation of synthetic movements with biological characteristics. This is however more than just an attempt to better fit data, Bennequin et al. [9] proposed a new theory of movement timing based on *geometrical invariance*. This includes important gemetric elements such as Cartan’s method of moving frames and arc-length parameterization, for the specific details the reader is referred to [9].

When it comes to synthetic motion, empirical laws such as the 2/3 power law, can be used as an indicator of ‘naturality’. For example, recently, Fischer et al. [17] used the 2/3 power law (as well as other biological laws) to test whether a muscoloskeletal model of the human upper limb displayed human-like characteristics after a series of reinforcement learning iterations.

Generation of synthetic movement with biological characteristics is known to be an important factor to facilitate human-robot collaboration [18] and there is evidence that, during human-machine physical interaction, nonbiological velocity patterns (i.e. different from a power law) implemented by the robot would result in larger interaction forces [19]. Specifically, the robot in [19] tracked an elliptic path while the human participant interacting with the robot was instructed to minimise the force applied by them on the robot (this information was supplied to the participant by providing online visual feedback of the force). Such a human-robot collaboration framework would benefit from the generalisation capability of tracing other curves with a biological velocity pattern, e.g., in industrial tasks like surface polishing, etc where contour tracing while applying a constant force is necessary. Thus, it is of interest for robots to generate human-like motion for a more effective human-robot collaboration. For a more comprehensive study on the technical approaches to generating human-like motion, the reader is referred to [20]. With this initial motivation for generating human-like motion, we next highlight other approaches to *movement synthesis*, followed by a gist of the proposed method and also compare with related works.

### 1.1 Related works

Continuous planar hand drawing movements have been studied widely to understand the predictive mechanisms in movement synthesis among humans [9, 10]. The 2/3 power law has been viewed as an emergent property of the motion planning principles employed by the brain [21]. The investigation of the origins of the 2/3 power law has yielded theories of movement synthesis using optimal control, i.e., (1) movement smoothness [22–24], (2) smoothness along a path [25] and (3) minimizing end-point variability [26].

Specifically, to appreciate the significance of movement synthesis in curve tracing, we highlight the difference between curve *tracking* and *tracing* first. We refer to curve tracing as the task of drawing a reference curve without any specification of the speed/time to reach a point on the curve, whereas curve tracking has to do with executing a time-sensitive trajectory around the curve. Methods to perform curve tracking include [27], where it was discussed that coupling two sinusoidal motions, i.e., of the form *x*(*t*) = *l*_*x*_ cos(*t*), *y*(*t*) = *l*_*y*_ sin(*t*) can produce an ellipse with the motion being empirically indistinguishable from the human-produced power-law. Within the same work, further investigations by choosing via-points at the extrema of ellipses with different eccentricities and employing variations of the minimum-jerk formulation (i.e., moving the visco-elastic body around an ellipse) help understand the time-dependence of the generation motion, since kinematic jerk is a time-dependent quantity. Within curve tracking literature, going beyond ellipses, the minimum-jerk model was also used to generate motion on a variety of curves [23] like the asymmetric figure of eight, cloverleaf, oblate limaçon, with inputs for each curve being a separate set of via-points, with selection criteria dependent on when velocity and acceleration changed sign in each dimension. These via-points were selected on all movement cycles in the human demonstrations of curve tracing.

In both the experiments above, the human subjects were however not given any cues as to the required speed of execution. In other words, the input was only the curve itself, i.e., a set of points. This emphasises the importance of geometry of curve, rather than specifying the speed to navigate the curve at. For an intuitive understanding, consider a human driving a car on a curvy road. The speed at which the human navigates a particular turn is determined by the local curvature (i.e., moving frame geometry) of the road at that point, rather than a pre-determined trajectory planned ahead of time. This is the intuition behind curve tracing. To the best of the authors’ knowledge, approaches to artificially generating human-like motion for curve tracing, independent of the time parameterization (i.e., curve tracking) are scarce. Our work proposes to use this idea of moving frame geometry to learn velocity-curvature patterns human exhibits during curve-tracing demonstrations. This is in contrast to the simulated trajectories using minimum jerk formulation between via-points on a curve proposed in other curve-tracing works [23, 24, 27]. The input in these works was a specification of speed and acceleration at each of those via-points. The moving frame we use in our method is a way to describe the curve independent of this time-parameterisation that the minimum jerk formulation requires. Secondly, in terms of generalisation capability to new curves, the minimum jerk formulation would require specification of both via-points and velocity/acceleration information. Using our method with geometric invariance, we are able to generalise to unknown curves without the need for any human demonstration on them. Furthermore, for complex curves, the number of via-points and their placements will affect the minimum-jerk solution. For example, it was shown how the presence of an excess via-point could induce an unnecessary loop in the trajectory [23], hence the choice of via-points is an extra requirement in this case. The use of geometric invariance to perform curve tracing avoids all these issues and allows for movement generation for curve tracing indefinitely, with just the geometric information of the curve, and no additional requirements such as via-points, etc.

In this work, we address the problems of i) learning from a kinematic demonstration of planar curve tracing and ii) generalizing to different (planar) geometries. As mentioned earlier, power laws have an inherently geometric flavor, and a novel aspect of the proposed approach is to formulate the curve-tracing problem in terms of *geometric invariants* and before applying (any) supervised learning methods. Learning geometric invariants, as opposed to rote memorization of a given trajectory, will be shown to be key to generalization.

In the next sections, we shall first recall basic geometric invariants of curves and then present a supervised learning method to fit subject-specific power laws. Finally, we shall show how geometric-invariant laws learned from specific subjects on specific curves can be generalized to different curves. More specifically, in Section 2, we recall that geometrically invariant quantities such as the curvature enable the reconstruction of a curve parametrically. This motivates our choice to train a learning machine using such invariant quantities, i.e., to learn the geometrical features of the curve itself. Our tool of choice for learning, i.e., the extreme learning machine (ELM), is detailed in Section 3. The setup and preparation of training data, along with the choice of geometric invariant quantities for input and output variables, is also presented. In Section 4, the experimental protocol for the various human subjects in this study and the evaluation criteria for benchmarking the performance of the corresponding trained ELMs with respect to the real human demonstration data is detailed with results. We conclude Section 4 with the discussion of the research gaps in this work and the future scope.

## 2 Geometric invariants behind curve-tracing

In this section, we shall first recall the geometric invariants for curves in the Euclidean plane. Notation-wise, vectors will be denoted in bold and will be represented as column arrays with components referred to some orthonormal Euclidean frame. The objective of this section is to identify the geometric invariants that help in the analysis of a curve and consequently help choose invariant observations to train a machine learning algorithm that can reproduce human-like movements when tracing similar or different curves.

### 2.1 Euclidean invariants of 2D curves

Following the convention for differential geometry in [28], consider a 2D curve as a smooth function $c:R\to R^{2}$ mapping a scalar parameter *p* ↦ ***c***(*p*) into the Euclidean plane. The first and second derivatives of ***c*** will be denoted as ***c***_*p*_ and ***c***_*pp*_. Consider now a re-parameterization of the same curve ***c***(*s*) ≔ ***c***(*s*(*p*)). By application of the chain rule, one can write all the higher-order derivatives as:

$$\begin{array}{c} c_{p}=c_{s}s_{p} \end{array}$$ (1)

$$\begin{array}{c} c_{pp}=c_{ss}s_{p}^{2}+c_{s}s_{pp}, \end{array}$$ (2)

where *s*_*p*_ and *s*_*pp*_ are the first and second derivative of *s*(*p*), respectively. Since the re-parametrization *s* is assumed to be strictly monotonic, the relations (1) and (2) can be inverted as:

$$\begin{array}{c} c_{s}=c_{p}/s_{p} \end{array}$$ (3)

$$\begin{array}{c} c_{ss}=\left(c_{pp}s_{p}-c_{p}s_{pp}\right)/s_{p}^{3}. \end{array}$$ (4)

The *velocity* vector ***c***_*p*_ is by definition tangent to the curve but its magnitude depends on the specific parameterization. By using the re-parameterization *s*(*p*) such that $s_{p}≔\left\|c_{p}\right\|\equiv \sqrt{\langle c_{p},c_{p}\rangle }$, where $\langle c_{p},c_{p}\rangle ≔c_{p}^{T}c_{p}$ is the *Euclidean inner product*, one can guarantee that ‖***c***_*s*_‖ = 1, from (3), and define the unitary vector ***t*** *tangent* to the curve as:

$$\begin{array}{c} t≔c_{s}=c_{p}/\left\|c_{p}\right\| \end{array}$$ (5)

The implicit assumption is that we are representing 2D vectors as column vectors with components relative to some *orthonormal frame*, therefore $c_{p}^{T}c_{p}$ (a scalar) corresponds to the sum of the squares of each component.

Given the tangent, the (unitary) *normal* to the curve ***n*** can be derived via a 90 degree counter-clockwise rotation J of the tangent vector, that is:

$$\begin{array}{c} n≔J\;t,\;\;\;\text{with}\;\;\;J≔[\begin{array}{cc} 0 & -1\\ 1 & 0 \end{array}]. \end{array}$$ (6)

Albeit not necessarily unitary, another vector orthogonal to the tangent is ***c***_*ss*_ and its length can be used to define the *Euclidean curvature* *μ*:

$$\begin{array}{c} \mu ≔\left\langle c_{ss},n\right\rangle =c_{ss}^{T}\;J\;t. \end{array}$$

This can be seen by differentiating $\|c_{s}{\|}^{2}=c_{s}^{T}c_{s}=1$ with respect to *s*, leading to $c_{ss}^{T}c_{s}=0$.

So far, all vectors were represented with respect to some (orthonormal) *space* frame. One can consider the family of *moving frames*, for example parameterized by *p*, with the origin at ***c***(*p*), on the curve, and its two axes always aligned with the tangent ***t*** and normal ***n*** vectors. The relationship between spacial and moving frames is captured by the matrix:

$$\begin{array}{c} M≔[\begin{array}{cc} t & n \end{array}]. \end{array}$$ (7)

It can be easily verified that ***M*** is a rotation matrix and in particular that ***M***^−1^ ≡ ***M***^*T*^. Using the definition of tangent vector (5), 〈***t***, ***t***〉 = 1 and hence $\langle t,n\rangle =t^{T}Jt=0$ and $\langle n,n\rangle ={\left(Jt\right)}^{T}Jt=1$. By denoting with a *prime* symbol (′) the variable represented with respect to a moving frame, one gets the following identities:

$$\begin{array}{ccc} t^{\prime } & ≔ & M^{T}t={\left[\begin{array}{cc} 1 & 0 \end{array}\right]}^{T}, \end{array}$$ (8)

$$\begin{array}{ccc} n^{\prime } & ≔ & M^{T}n={\left[\begin{array}{cc} 0 & 1 \end{array}\right]}^{T}, \end{array}$$ (9)

$$\begin{array}{ccc} \mu^{\prime } & ≔ & \left\langle c_{ss}^{\prime },n^{\prime }\right\rangle =\left\langle c_{ss},n\right\rangle =\mu . \end{array}$$ (10)

The latter is a particular instance of the invariance of the inner product. The curvature is such an invariant and knowledge of the curvature at any point of a curve is sufficient to reconstruct the entire curve, *modulo a translation and a rotation* [29].

**Fig 1:**
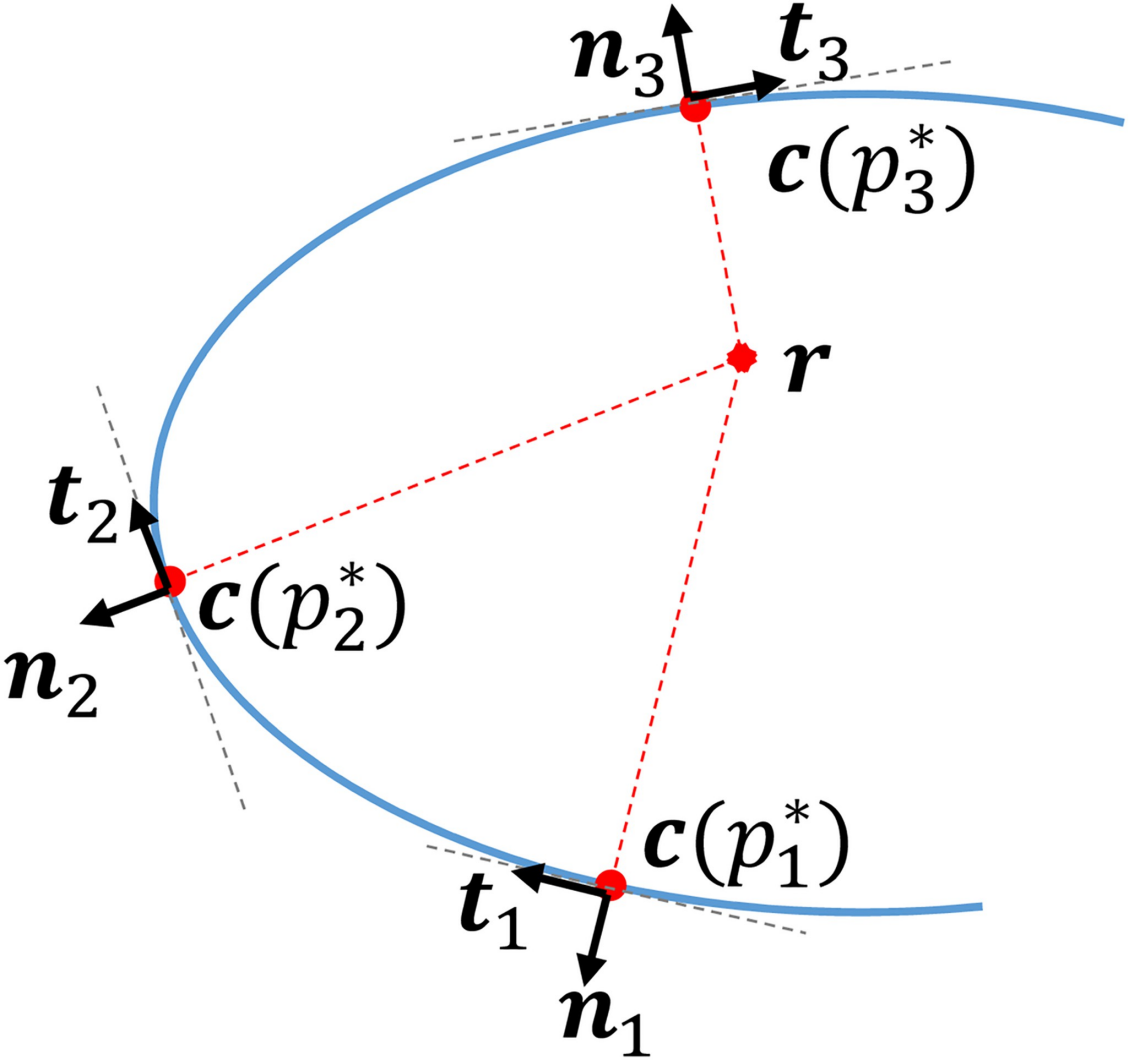
Points on a curve extremizing the distance to a point *r*. When these points are interior, the tangent at those points is always perpendicular to the segment joining ***r*** to the point itself.

Another important invariant is the *distance* of a given point ***r*** to the curve, see Fig 1. For any point ***c***(*p*) on the curve, one can evaluate the (squared) distance ‖***r*** − ***c***(*p*)‖^2^ and determine the parameter value p* as its minimizer within a certain interval I⊂R:

$$\begin{array}{c} p^{*}≔\underset{p\in I}{\text{argmin}}\;{\left\|r-c\left(p\right)\right\|}^{2}. \end{array}$$ (11)

As clarified later, one might not always be interested in a global minimum, and, by properly selecting the interval *I*, it is possible to exclude unwanted minima. Moreover, whenever p* in (11) is an interior point, the vector ***r*** − ***c***(p*) is normal to the curve i.e., 〈***t***, ***r*** − ***c***(p*)〉 = 0.

One of the main ideas of this paper is to use geometric invariants such as the curvature to train a learning machine. In the specific case of tracing tasks, we expect the machine to learn the intrinsic geometrical features of a curve, independently of possible rotations and translations of the same curve in the workspace.

## 3 Geometric-invariant learning

In this work, we choose as learning paradigm a class of neural networks called *extreme learning machines* (ELMs) [30] since they have been shown to have the same universality approximation properties as neural networks [31], but they are much easier to train. In contrast with conventional neural networks, where the weights need to be estimated iteratively, the training of ELMs requires only a single linear regression step for the output layer parameters, while the rest of the parameters are randomly initialised. An additional regularization step is also needed to mitigate the potential ill-conditioning of the regression problem. To be more specific, ELMs are defined by the following set of equations:

$$\begin{array}{c} \left(ELM\right)\{\begin{array}{c} x_{t}=\sigma (cCz_{t}+\zeta ),\\ y_{t}=Wx_{t}+b, \end{array} \end{array}$$ (12)

where at each sampling time *t*, the neuronal activity is represented by an internal state vector $x_{t}\in R^{N_{x}}$ driven by an input vector $z_{t}\in R^{N_{z}}$. A nonlinear function *σ*(⋅) is assumed to be the hyperbolic tangent (tanh(⋅)) that acts elementwise, the parameter $c\in R^{+}$ is responsible for scaling of the matrix $C\in R^{N_{x}\times N_{z}}$ which together with the vector $\zeta \in R^{N_{x}}$ is randomly generated. The output of the ELM is represented by the vector $y_{t}\in R^{N_{y}}$ that is obtained as an affine transformation of the state vector ***x***_*t*_ with $W\in R^{N_{y}\times N_{x}}$ and $b\in R^{N_{y}}$ the constant matrices.

We emphasize that we shall be using the ELM with the same fixed ***C*** and ***ζ***, in order to enable comparison across the ELMs trained on data collected from different human subjects. More specifically, the randomly generated ELM is kept fixed all along our experiment, and it is only the scaling parameter *c*, readout matrices $W\in R^{N_{y}\times N_{x}}$ and $b\in R^{N_{y}}$ as well as the regularization constant λ_*rdg*_ used in the solution of the regression problem that are tuned for each different subject in the study. This means that the ELM encodes the generic tracing task and the readout parameters account for the specificities and tracing style of the individual subjects.

### 3.1 ELM setup and data preparation

The starting point in setting up an ELM is choosing the dimension of the input vectors (*N*_*z*_), the output vectors (*N*_*y*_) and, more importantly, of the internal state vectors or neurons (*N*_*x*_). Note that the input and output vectors are determined by the problem itself, i.e., the mapping that needs to be learned. More explicitly, *N*_*z*_ is the number of available explanatory covariates, and *N*_*y*_ is the number of dependent variables. As an example, *N*_*z*_ could be the set of observations about the world and *N*_*y*_ the number of controls, in the case of a robotic task.

For the scenarios of interest in this work, the purpose of the ELM is to learn the unknown dynamics of a given controlled physical process, such as the curve tracing task, based on historical subjects-generated tracing data. In this sense, the most elementary task that one may think of is using as inputs *m* sequences $\left\{{\tilde{z}}_{1},{\tilde{z}}_{2}\ldots ,{\tilde{z}}_{m}\right\}$ containing the positions in time and space of the pencil as driven by the subjects and to train the ELM in order to produce outputs $\left\{{\tilde{y}}_{1},{\tilde{y}}_{2}\ldots ,{\tilde{y}}_{m}\right\}$ associated to each of *m* samples (or *measurements*) that predict the tracing, that is, ***y***_*t*_ ≡ ***z***_*t*+1_.

We shall see later on in Section 3.4 that we might decide to augment the historical input state with additional information, which will, of course, increase the input dimensionality. When it comes to the output vector, apart from predicting the next state given the current input, one might want to try to predict the velocity of the tracing, which also leads to an augmentation of the output dimensionality. In both cases, it is clear that *N*_*y*_, *N*_*z*_ are specified by the input/output dimensionality of the dynamic process that one is trying to learn.

The dimension of the state space *N*_*x*_ is an architecture hyperparameter that determines the complexity of the model. Even though approximation bounds have been formulated [31] for this machine learning paradigm in which *N*_*x*_ appears as a variable, the lack of knowledge about the stochastic properties of the data-generating process at hand makes it very difficult to use them. In this work and after a trial and error procedure, the number of neurons has been fixed at *N*_*x*_ = 100.

### 3.2 ELM training

Given input and output sequences of historical data, training an ELM consists in determining a matrix ***W*** and a vector ***b*** such that, when the input sequence of historical data is fed into the ELM, the outputs ***y***_*t*_ produced by (12) approximate well the given historical target sequence of outputs. Before training begins, it is useful to prepare the following historical data matrices

$$\begin{array}{c} \left(training\;set\right)\{\begin{array}{ccc} {\tilde{Z}}_{trn} & ≔ & \left[\begin{array}{cccc} {\tilde{z}}_{1} & {\tilde{z}}_{2} & \cdots & {\tilde{z}}_{m} \end{array}\right]\\ {\tilde{Y}}_{trn} & ≔ & [\begin{array}{cccc} {\tilde{y}}_{1} & {\tilde{y}}_{2} & \cdots & {\tilde{y}}_{m} \end{array}]. \end{array} \end{array}$$ (13)

For each ${\tilde{z}}_{t}$, compute ***x***_*t*_ based on the first equation in (12) and construct the matrix of internal states

$$X_{trn}≔[\begin{array}{cccc} x_{1} & x_{2} & \cdots & x_{m} \end{array}].$$

The ELM training consists in solving the ridge regularized linear regression problem that minimizes the difference between historical output sequence ${\tilde{Y}}_{trn}$ and sequence of outputs ***Y***_*trn*_ = ***W*** ***X***_*trn*_ + ***b*** as predicted by the ELM (12), i.e.

$$\begin{array}{c} \left(W^{*},b^{*}\right)=\underset{W,b}{\text{argmin}}\;\{\|{\tilde{Y}}_{trn}-Y_{trn}{\|}_{2}^{2}+\lambda_{rdg}{\left\|W\right\|}_{\text{Fro}}^{2}\}. \end{array}$$

The parameter λ_*rdg*_ > 0 determines the strength of the ridge penalty term $\lambda_{rdg}{\left\|W\right\|}_{\text{Fro}}^{2}$ that is introduced to solve potential ill-conditioning problems in the covariance matrix of the internal states. The solution to this optimization problem can be written down in a closed form:

$$\begin{array}{c} W^{*}={\left(X_{trn}X_{trn}^{T}+\lambda_{rdg}I_{N_{x}}\right)}^{-1}X_{trn}{\tilde{Y}}_{trn}, \end{array}$$ (14)

$$\begin{array}{c} b^{*}=\frac{1}{m}{\tilde{Y}}_{trn}1_{m}-\frac{1}{m}W^{*}X_{trn}1_{m}, \end{array}$$ (15)

where **1**_*m*_ ≔ [1, …, 1]^*T*^ is the *m*-dimensional vector with ones at each entry. The training error or goodness of fit is measured by computing the mean square norm of the difference between ${\tilde{Y}}_{trn}$ and ***Y***_*trn*_, the latter being computed using the parameters obtained in (14) and (15). The universality properties of ELMs proved in [31] guarantee that this error can be made arbitrarily small by choosing *N*_*x*_ sufficiently large.

### 3.3 ELM testing and forecasting

A standard problem in machine learning consists in finding the right model complexity (in our case, tuned by the number of neurons *N*_*x*_) that minimizes the so-called expected loss or the generalization error. We recall that both too-large and too-small training errors may imply an unsatisfactory generalization error, and hence minimizing the training error for a given sample size does not imply optimal generalization error. We emphasize that we assume no knowledge about the joint distribution of input and target variables and hence do not have access to the generalization error. We hence use a different set of historical data, *testing set*, and use the testing error to assess the performance of the ELM on the learning task of interest.

Specifically, given input and output sequences of *s* measurements, one forms the following matrices

$$\begin{array}{c} \left(testing\;set\right)\{\begin{array}{ccc} {\tilde{Z}}_{tst} & ≔ & \left[\begin{array}{cccc} {\tilde{z}}_{m+1} & {\tilde{z}}_{m+2} & \cdots & {\tilde{z}}_{m+s} \end{array}\right]\\ {\tilde{Y}}_{tst} & ≔ & [\begin{array}{cccc} {\tilde{y}}_{m+1} & {\tilde{y}}_{m+2} & \cdots & {\tilde{y}}_{m+s} \end{array}], \end{array} \end{array}$$

where for each ${\tilde{z}}_{t}$ in the testing set (*m* < *t* ≤ *m* + *s*), the trained ELM in (12) is used to generate a sequence of predicted outputs ***y***_*t*_, conveniently stored in a matrix

$$\begin{array}{c} Y_{tst}≔[\begin{array}{cccc} y_{m+1} & y_{m+2} & \cdots & y_{m+s} \end{array}]. \end{array}$$

The testing error of the ELM on a separate sample than the one used in the training is measured by computing the mean square norm of the difference between ${\tilde{Y}}_{tst}$ and ***Y***_*tst*_, the latter being computed using the parameters obtained in (14) and (15). Additionally, an optimal ridge regularization constant λ_*rdg*_ needs to be selected. This is done in practice by reserving a third disjoint sample (we call it *validation sample*) on which we choose the value of λ_*rdg*_ that minimizes the validation error.

**Fig 2:**
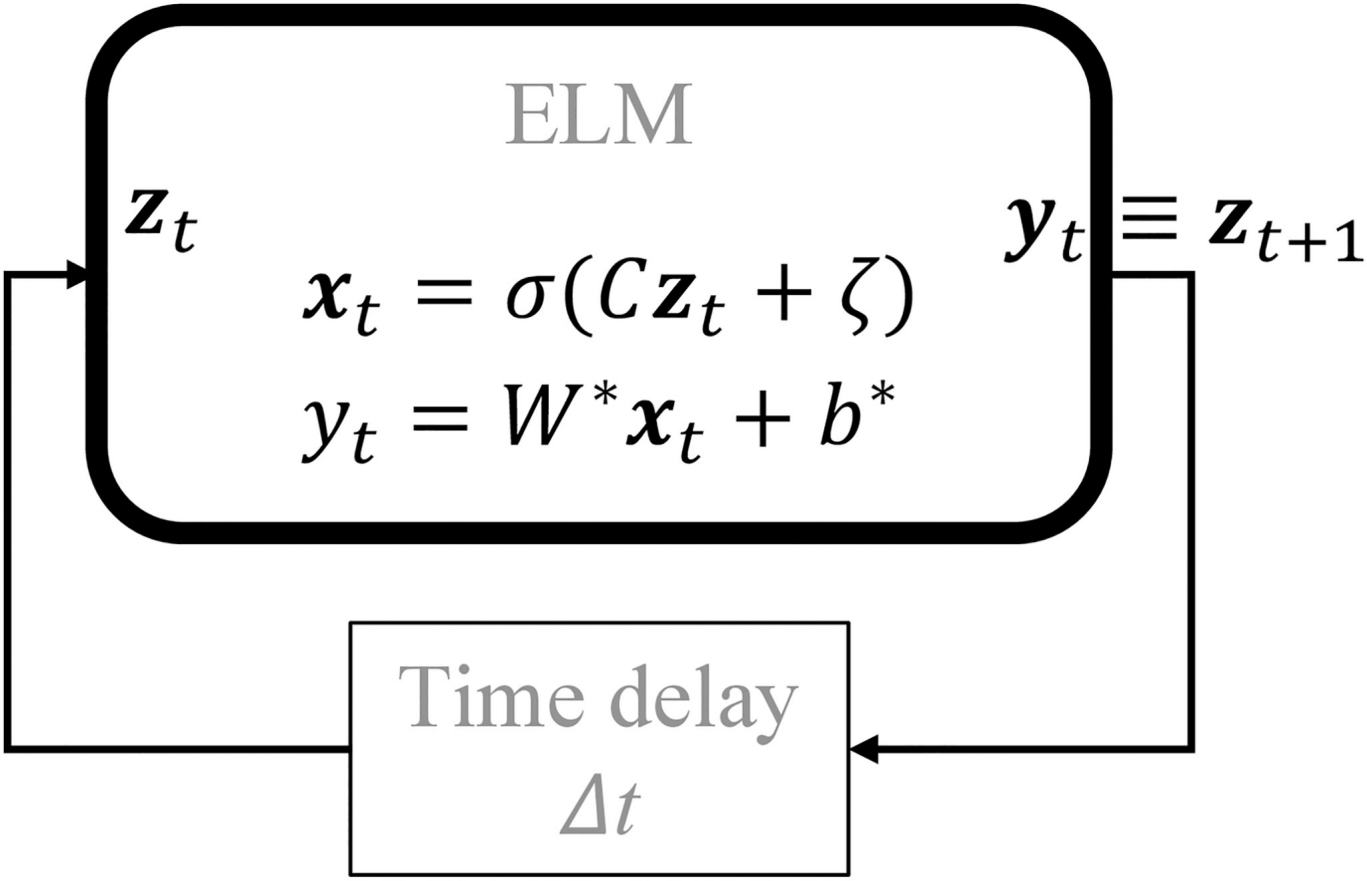
Self-looped configurations of a trained ELM to forecast discrete-time dynamical systems. Note that Δ*t* in a discrete-time experiment corresponds to the sampling rate.

An ELM trained for the one-step ahead prediction task can be *self-looped* to forecast the output in an autonomous manner as in Fig 2. The testing error of the ELM in this autonomous regime can be also measured.

This is the data generation process that will be used in Eq 19 in the next subsection when preparing the training set for learning the curvature-velocity relationship from human tracing data.

### 3.4 Invariant training of ELMs from 2D curve-tracing data

Consider two curves on the Euclidean plane: the first being a given analytical 2D curve *p* ↦ ***c***(*p*) parameterized by some (real) parameter *p* as in Section 2.1 that represents the curve that the subject needs to trace. The second curve is a discrete sequence of points $t_{i}\mapsto r_{t_{i}}$ representing the physical motion, with respect to time *t*_*i*_ ∈ {1, 2, …, *m*} of an agent tasked to trace the given analytical curve. We shall assume that both curves are expressed in coordinates relative to the same orthonormal reference frame and that, as a result of a measuring process, the physical motion is only available as a dataset consisting of samples of positions $r_{t_{i}}$ and velocities ${\dot{r}}_{t_{i}}$ collected at times *t*_*i*_ = 1, 2, …, *m*. It may be noted that the index *i* refers to the discrete sample in the dataset. For ease of notation, henceforth we shall omit the notation *t*_*i*_ and simply refer to positions as ***r***_*t*_ and velocities as ${\dot{r}}_{t}$.

Training starts with the definition of the training set (13) and, to this end, a complete set of information about the tracing task and the curve would include, at any time *t* = 1, 2, …, *m*, the agent’s position ***r***_*t*_ and velocity ${\dot{r}}_{t}$ as well as the closest point on the curve ***c***(p*), the tangent ***t***, the normal ***n*** and curvature *μ*, all evaluated at p*, where p* is determined by (11). We notice that in this case the training set depends on the specific reference frame used to define the curve and collect measurements.

In this paper we are interested in defining a *reference-invariant learning* from the available data. Given an ELM, our goal is to design an invariant training procedure that is *independent of any specific choice of reference frame used to define the curve and make measurements*.

In order to achieve this purpose, we propose to represent the available data in terms of *moving frame coordinates* before forming the training set (13). Specifically, at any time *t* = 1, 2, …*m*, we shall consider the (unique) orthonormal frame centered at ***c***(p*) and aligned with ***t*** and ***n***. Algebraically, this corresponds to computing the following *linear* transformations:

$$\begin{array}{ccc} r_{t} & \mapsto & r_{t}^{\prime }=M^{T}\left(r_{t}-c\left(p^{*}\right)\right), \end{array}$$ (16)

$$\begin{array}{cc} {\dot{r}}_{t} & \mapsto {\dot{r}}_{t}^{\prime }=M^{T}{\dot{r}}_{t},\\ c(p^{*}) & \mapsto c^{\prime }\left(p^{*}\right)=M^{T}\left(c\left(p^{*}\right)-c\left(p^{*}\right)\right)\equiv 0,\\ t & \mapsto t^{\prime }\equiv {\left[1\;0\right]}^{T},\\ n & \mapsto n^{\prime }\equiv {\left[0\;1\right]}^{T},\\ \mu & \mapsto \mu^{\prime }\equiv \mu , \end{array}$$ (17)

where the transformed (primed) quantities are invariant by definition with an added computational advantage that ***c***′(p*), ***t***′, and ***n***′ are *constant* and can hence be removed from the training set. Finally, as remarked earlier in relation to the optimality of p* in (11), the vector ***r***_*t*_ − ***c***(p*) in (16) is necessarily *normal to the curve* when p* is an interior point. This implies that the first component of $r_{t}^{\prime }$, namely 〈***t***, ***r***_*t*_ − ***c***(p*)〉, is always zero while the second component is an *invariant* which can be used to define the task *signed error*

$$\begin{array}{c} e_{t}≔\left\langle n,r_{t}-c\left(p^{*}\right)\right\rangle \equiv \left\langle n^{\prime },r_{t}^{\prime }\right\rangle =e_{t}^{\prime } \end{array}$$ (18)

and which can be used to replace $r_{t}^{\prime }$.

In conclusion, we propose to form the training vectors ${\tilde{z}}_{t}\in R^{3}$ in (13) for an ELM based on the following definition:

$$\begin{array}{c} {\tilde{z}}_{t}=\frac{1}{{\hat{\sigma }}_{z_{trn}}}\;\left(z_{t}-{\bar{z}}_{trn}\right),\;\;\text{with}\;\;z_{t}=[\begin{array}{c} e_{t}^{\prime }\\ \mu_{t}^{\prime }/\mu_{0}\\ p_{t}^{*}/p_{0} \end{array}],\;{\tilde{y}}_{t}=\frac{1}{{\hat{\sigma }}_{y_{trn}}}\left({\dot{r}}_{t}^{\prime }-{\bar{y}}_{trn}\right), \end{array}$$ (19)

and where at any time *t* = 1, 2, …*m*, an optimal p* is evaluated via (11), $e_{t}^{\prime }$ is computed as in (18), *μ*′ is computed as (10), ${\dot{r}}_{t}^{\prime }$ is computed as in (17), while the sample mean ${\bar{z}}_{trn}$ and the sample standard deviation ${\hat{\sigma }}_{z_{trn}}$ of the training set of observations and, the sample mean ${\bar{y}}_{trn}$ and the sample standard deviation ${\hat{\sigma }}_{y_{trn}}$ of all training speeds are used to demean and standardize the training dataset. In order to ensure that the inputs to the ELM are within the range of the activation function for each neuron, we use normalisation factors *μ*_0_ = 50 *m*^−1^ and *p*_0_ = 10 *rad* to enforce that the curvature and the parameter p* fall in the interval [−1, 1].

## 4 Learning subject-specific power laws

**Fig 3:**
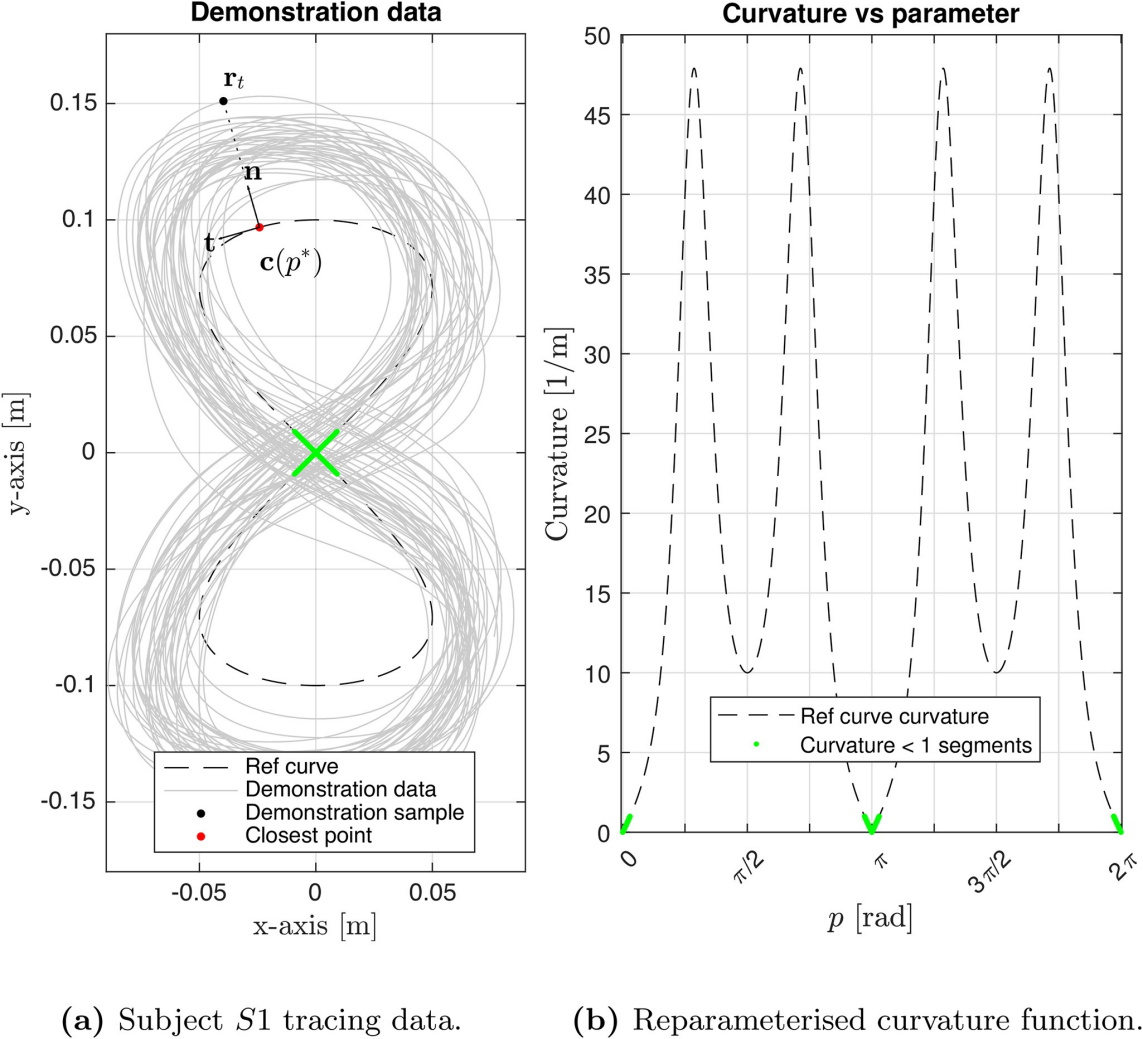
(**a**) The figure of eight was provided as the reference curve to the subjects to perform tracing demonstrations on and the training data for the ELM was prepared by selecting the data-points where the speed was non-zero. (**b**) Omitted regions with the curvature close to zero are marked in green.

In this section, the proposed theoretical framework is applied to a set of experimental data. We asked healthy subjects to perform a planar tracing task (shown in Fig 3(a)) and, for each subject, we train a randomly initialized ELM.

The objective of the study is to assess the similarities between the “motor style” of each human subject and the synthetic motion generated by a trained ELM in self-looped mode as in Fig 2.

### 4.1 Human motion and power laws

In this subsection, we discuss the data collection and the power law model which is used to explain the individual motor styles of human subjects. We provide the details regarding the setup of the experiment, data preprocessing, and fitting.

#### Power law of human motion

In this work we consider a 2-geometry model for evaluating a power law as follows:

$$v=\{\begin{array}{cc} v_{c}, & \text{if}\;\kappa <\kappa_{c}\\ v_{c}{\left(\kappa /\kappa_{c}\right)}^{-\beta }, & \text{otherwise}, \end{array}$$

where, the *critical* speed and curvature are denoted by *v*_*c*_ and *κ*_*c*_, respectively, and *β* is the power law slope. It has been established that humans trace curve segments with low curvature (*κ* < *κ*_*c*_) with near constant speeds (*v*_*c*_) and the power law is thus characterised by these quantities (*κ*_*c*_ and *v*_*c*_) [9]. To evaluate the power law, we fix *β* = 1/3 [9] before obtaining *v*_*c*_ and *κ*_*c*_ through a piecewise logarithmic linear regression on the tracing data using the following model:

$$\begin{array}{c} \text{log}\left(v\right)=\text{log}\left(v_{c}\right)+\beta \left(\text{log}\left(\kappa_{c}\right)-\text{log}\left(\kappa \right)\right)\frac{1+\text{sign}(\kappa -\kappa_{c})}{2}, \end{array}$$ (20)

where *κ* is evaluated at each *t* as:

$$\begin{array}{c} \kappa =\frac{|\dot{x}\ddot{y}-\ddot{x}\dot{y}|}{{\left({\dot{x}}^{2}+{\dot{y}}^{2}\right)}^{3/2}} \end{array}$$ (21)

and *v* is the 2-norm of the vector ${\left[\;\dot{x},\dot{y}\;\right]}^{T}$ with *x* and *y* as in Fig 3(a).

#### Subjects and protocols

Ten healthy subjects (NTU-IRB Reference: IRB-2018-07-043) were asked to perform a tracing task on a planar robot, called H-Man [32], which is a table-top, portable planar robot. This robot was used only as 2D sensing device, with the motors always off. For the purpose of this study, the participants were instructed to trace out the figure of eight using the passive robot as accurately as possible with the following geometric definition:

$$c(p)=[\begin{array}{c} 0.05\;\text{sin}(2p)\\ 0.1\;\text{sin}(p) \end{array}].$$

**Fig 4:**
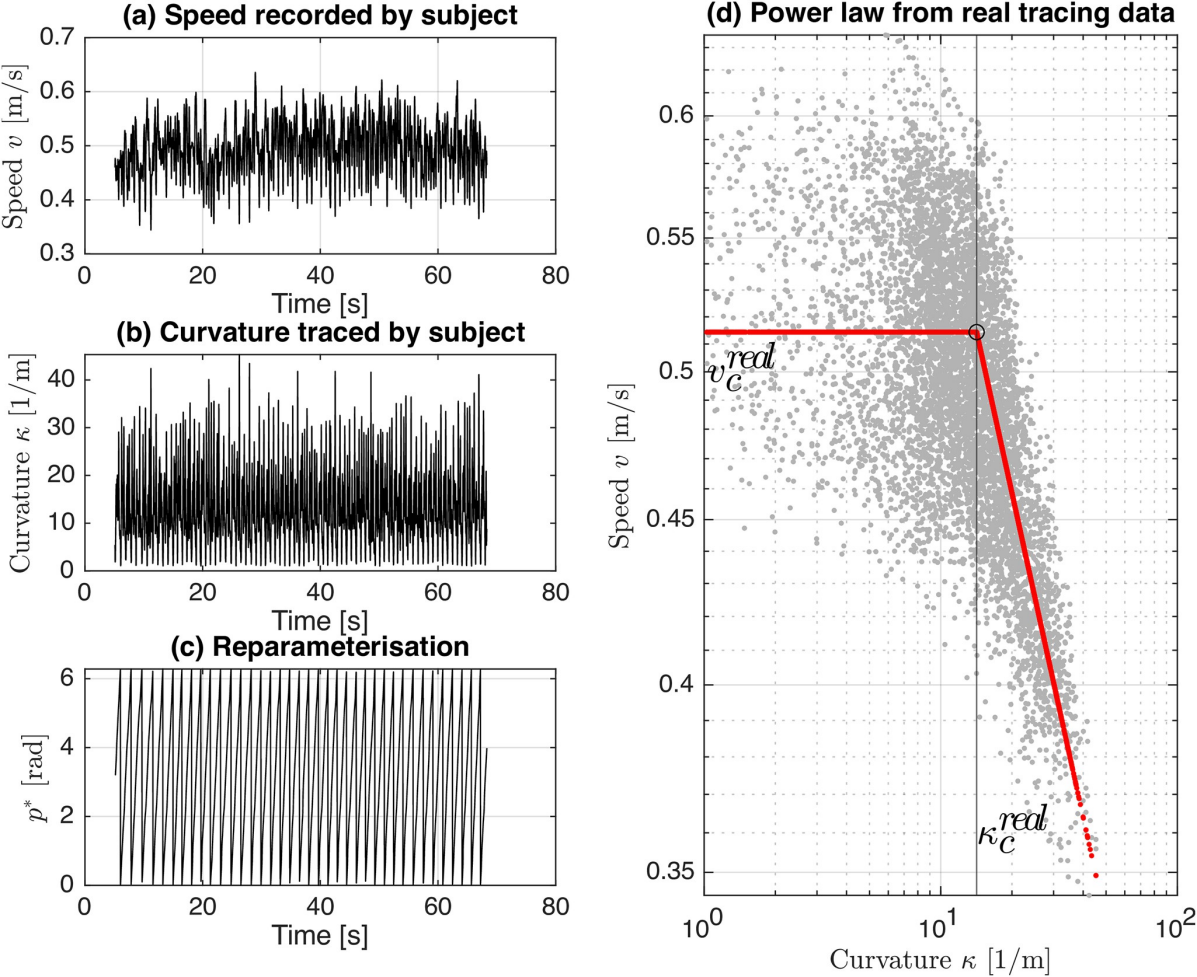
*S*1 tracing data for the figure of eight (the reference curve geometry) showing (a) speed *v*, (b) curvature *κ*, (c) Euclidean reparameterisation of tracing data *t* ↦ p* to yield the pairs {***r***_*t*_, ***c***(p*)} satisfying (11), and (d) the piecewise linear power law estimated for *S*1 tracing data {***r***_*t*_}, yielding critical speed $v_{c}^{real}$ and curvature $\kappa_{c}^{real}$.

Tracing data ${\left\{r_{t}\right\}}_{t=1}^{m}$ was collected for individual subjects and downsampled to a uniform sampling rate of 100 *Hz*, i.e., Δ*t* = 1/100 *sec*. This data was preprocessed and subsequently the piecewise linear power law as in (20) and (21) is fit to the data. Fig 4 demonstrates the reference geometry, estimated speed, and curvature, along with the tracing data for subject *S*1.

#### Preprocessing of human tracing data

The tracing data is preprocessed through a set of steps before evaluating the power law and deriving the critical speed *v*_*c*_ and curvature *κ*_*c*_, which serve to individually identify each subject. First, the scalar speed *v* and curvature *κ* are evaluated for each time sample using (21). The tracing data is then additionally filtered to remove the stationary instances by applying a zero-speed threshold. This is also important to avoid evaluating the logarithmic power law when speed or curvature is close to zero. Hence a curvature threshold of 1 *m*^−1^ is also applied to avoid these instances (see Fig 3(b)).

#### Estimation of human power law

The power law characteristics (the critical speed and curvature) are calculated for each subject’s tracing data using the model defined in (20) and labelled $\kappa_{c}^{real}$ and $v_{c}^{real}$—to attribute to data obtained from *real* human trials. All the codes are implemented in Matlab (2022b), are available on Github, and the computations are run on a MacBook Pro with 2,9 GHz 6-Core Intel Core i9 processor and 32 GB 2400 MHz DDR4 memory.

The data points and the estimated power law characteristics $v_{c}^{real}$, $\kappa_{c}^{real}$ are shown in Fig 4(d) for subject *S*1 and listed in Table 1 for all the ten subjects in our study.

**Table 1:** Piecewise power law quantities evaluated from tracing data of ten subjects.

	Human power law parameters		ELM power law parameters	
	$v_{c}^{REAL}$ (m/s)	$\kappa_{c}^{REAL}\left(1/m\right)$	$v_{c}^{ELM}\left(m/s\right)$	$\kappa_{c}^{ELM}\left(1/m\right)$
S1	0.514	14.181	0.489	19.001
S2	0.268	15.120	0.245	22.998
S3	0.244	15.525	0.223	22.896
S4	0.148	26.408	0.145	21.699
S5	0.347	16.155	0.333	15.236
S6	0.144	18.891	0.141	16.680
S7	0.136	22.812	0.130	18.487
S8	0.219	19.419	0.201	22.031
S9	0.205	21.123	0.195	22.015
S10	0.094	20.526	0.096	10.742

### 4.2 Generalization: Learning to trace like a human

In this subsection we discuss the implementation of the extreme learning machine (ELM) used to learn the motor style of individual subjects and generalize on the unseen in the training curves.

#### ELM architecture

We implement the ELM as in (12) with *N*_*x*_ = 100 neurons. The bias vector ***ζ*** is chosen to be a zero vector while the connectivity matrix ***C*** is sampled according to the law U{-1,1}.

#### ELM hyperparameter tuning

Both the scaling parameter $c\in R^{+}$ and the regularization $\lambda^{rdg}\in R^{+}$ are tuned for each individual subject separately. More explicitly, these hyperparameters are chosen such that they minimize the mean square error of the autonomous *l* = 100 steps operation of the ELM on a set of validation samples constructed reserving some part of the total training data. For each subject, we construct a set of indices S with step 10 starting from 10 and to $\left\lfloor \frac{1}{4}\left(m-l\right)\right\rfloor$, where *m* is the length of the total training sample for a given individual subject. For each index i∈S a particular instance of the *i*-th training sample with the length $\left\lfloor \frac{3}{4}\left(m-l\right)\right\rfloor -\frac{1}{2}l$ is used to obtain the estimated readout ***W***_*i*_ and intercept ***b***_*i*_. Next, the associated validation error *E*_*i*_ is measured as the mean squared distance between the result of the autonomous run of the ELM for *l* steps and the corresponding *l*-long validation sample. The optimal scaling parameter *c* and the ridge penalty strength λ^*rdg*^ are obtained as minimizers of the total validation error $\frac{1}{\left|S\right|}\sum_{i\in S}E_{i}$. Once the optimal hyperparameters are found, the total *m*-long training sample is used to estimate the readout matrix and the intercept for the ELM following the scheme presented in Subsection 3.4.

#### Data preparation for ELM

For each point in the tracing data ${\left\{r_{t}\right\}}_{t=1}^{m}$, the closest point on the curve in the Euclidean metric was computed using (11) to obtain the corresponding point $p_{t}^{*}$. The mapping $t\mapsto p_{t}^{*}$ for the experimental data is shown in Fig 4. A single point from the tracing data, ***r***_*t*_, and the corresponding $c(p_{t}^{*})$ are indicated as an example in Fig 3. This reparameterisation allows to evaluate moving frame quantities required to define the invariant mapping that will be used to train the ELM.

*Remark*. It must be noted that the initialisation of the parameter p* during this step is important and must be done manually to reflect the direction of movement. For instance, for the figure-of-eight curve, the Euclidean reparameterisation at the center of the curve may yield 0 (when the direction of movement is northeastward) or *π* (when the direction of movement is southeastward). This is a step that requires to be handled manually as the initial value depends on the way the human subject traces the curve, i.e., whether the human tracing is clockwise or counterclockwise. After initialising p*(0) manually as described, the choice of the interval *I* in the local minimisation algorithm discussed in (11) is chosen to be ±*π*/4 to limit the locality of search and not produce unwanted minima. This becomes especially important in curves that have overlapping or adjoining loops.

#### The tracing data of the extreme learning machine

**Fig 5:**
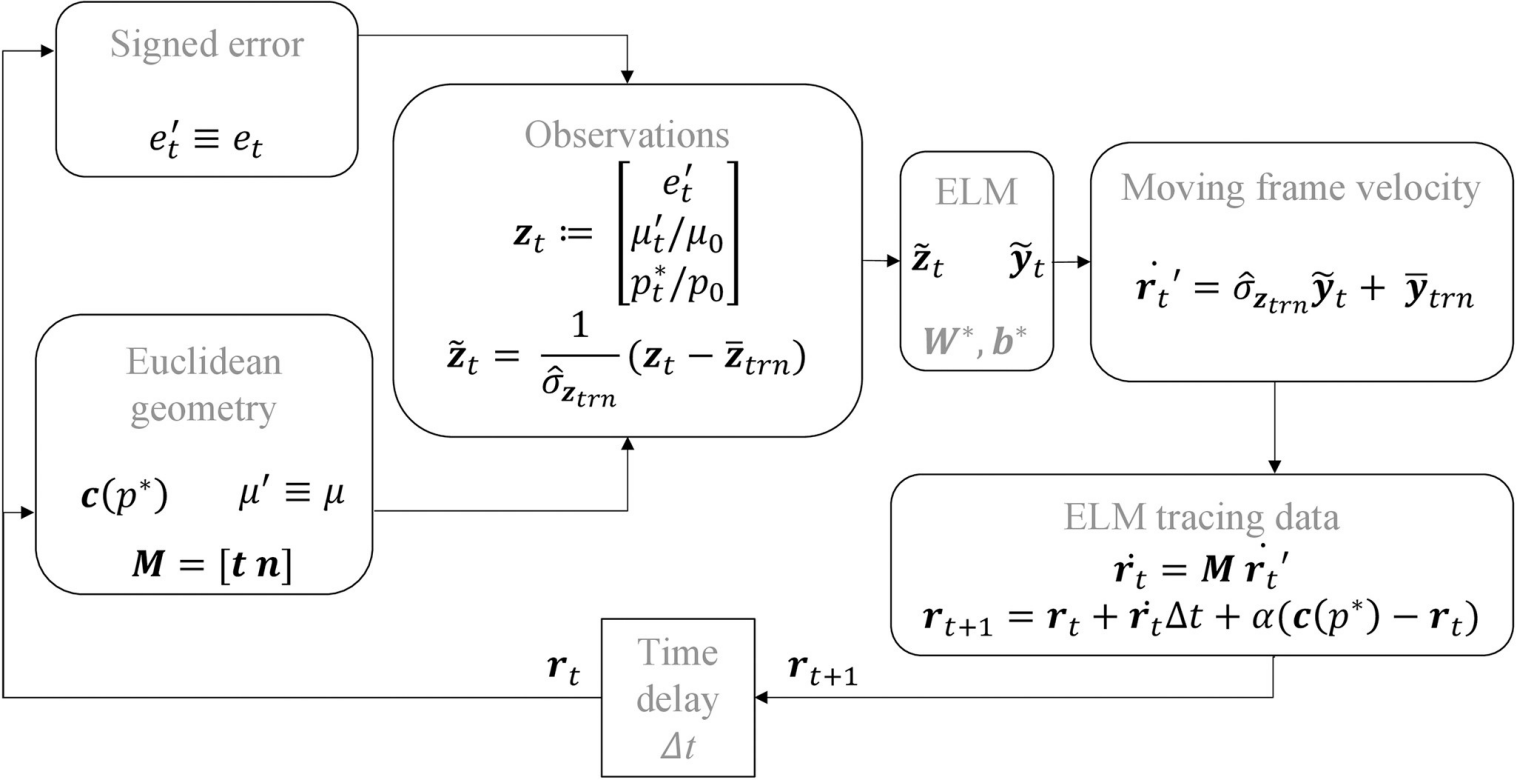
*Self-looping ELM*: Generalisation of the tracing task is based on the observation set *z*_*t*_ constituting of the signed error evaluated at the current point with respect to the reference geometry *c*(p*) after the reparameterisation step is done, the Euclidean curvature in moving frame *μ*′ and the parameter p*—the same structure as the training set data $\left\{{\tilde{z}}_{t}\right\}$ (Eq 19). The trained ELM predicts the moving frame velocity ${\dot{r}}_{t}$ that the tracing task requires at time *t*. The instantaneous spatial frame position of the tracing task is updated to ***r***_*t*+ 1_ with an additional force field *α*(***c***(p*) − ***r***_*t*_) imposed to ensure stability, where *α* = 0.05 was heuristically chosen.

**Fig 6:**
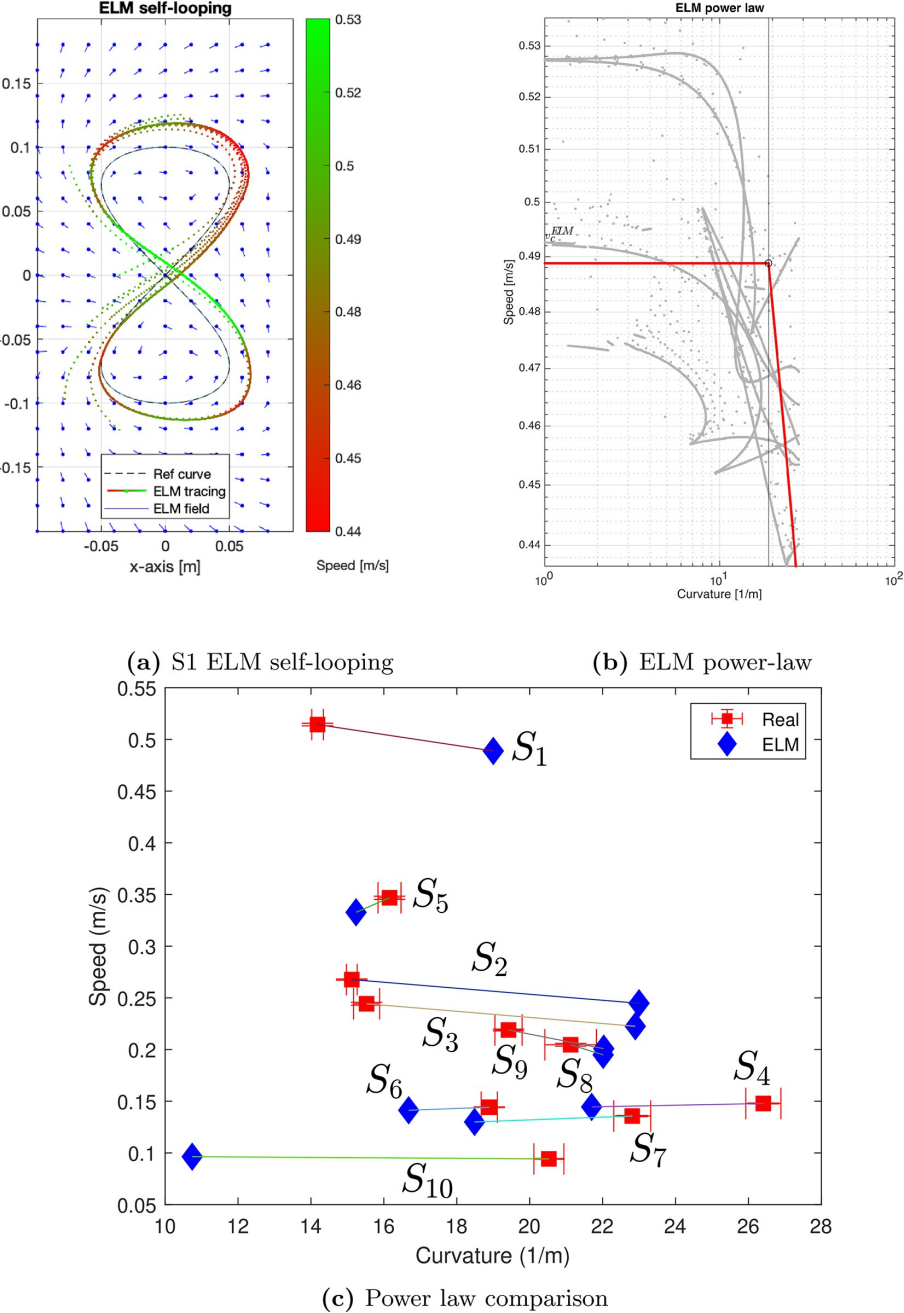
(**a**) The ELM self-looping behavior may be observed as going slow at the corners and picking up speed along the straighter segments, plotted against the reference curve geometry and the ELM-induced velocity field around the reference geometry. (**b**) Piece-wise power law evaluated for ELM data. (c) Comparison of the ELM performance with the human demonstration data based on the piecewise power law.

In order to assess the ability of the ELM to learn the motor style of each human subject, we use the same power law model as presented in (20) and (21). After the training of the ELM the self-looping ELM shown in Fig 5 is initialized at random at an arbitrary point in the vicinity of the reference geometry picked from the training dataset and tasked to self-loop with the criteria defined in Fig 2 for a duration of 100 seconds, i.e., 100/Δ*t* = 10000 steps. This process was repeated with different initial points for 10 trials to test the robustness of ELM tracing. The tracing performance resulting from this process for the ELM trained on subject *S*1 data, is shown in Fig 6(a) in addition to ELM-induced *velocity field*, i.e the output velocity of the ELM network for a given input (grid on the 2D plot).

#### Preprocessing of the ELM tracing data

Similar to human tracing, the dataset is preprocessed through a set of steps before evaluating the power law and deriving the critical speed *v*_*c*_ and curvature *κ*_*c*_ for ELM tracing instances corresponding to individual subjects. Analogously to the human data, the scalar speed *v* and curvature *κ* are evaluated for each time sample using (21). A zero-speed threshold and a curvature threshold of 1 *m*^−1^ are applied to the data. In addition to the steps adopted for the human data, we apply the trimming of the extreme values (outliers) to the ELM autonomous tracing data via keeping only those observations for which the curvature values are within the 85-th percentile.

#### Estimation of the ELM power law

Parameters of the same model for the power law which is used for human subjects and which is spelled in (20) and (21) are estimated using the preprocessed ELM tracing data. Due to the fact that the piecewise linear regression provides an upward biased estimate of the critical curvature, we modify the estimation procedure to address this issue. More precisely, we add a penalty on the curvature value and choose the penalty strength using cross-validation. For that purpose, we randomly split the ELM tracing data into 80%-long training sample and hold out 20% of the remaining data for validation. We chose the optimal penalty strength as a minimizer of the validation error on the held-out validation set. We label the power law critical curvature and critical velocity characteristics associated with the ELM as $\kappa_{c}^{ELM}$ and $v_{c}^{ELM}$, respectively. We showcase the results of the power law estimation for subject *S*1 in Fig 6(b).

#### Generalization with ELMs for the learned curve

We now illustrate the ability of ELMs to learn subject-specific power laws comparing the power law characteristics *v*_*c*_ and *κ*_*c*_ for the *real* and *ELM* cases. The results of this comparison presented in Table 1. We note that the ELM performance depends on the variability in the intrinsic speed of the subjects. The more regular the training data is (in terms of the speed in a single pass around the curve across multiple loops), the closer the performance of ELM to the power law obtained from real tracing data is.

#### Generalization with ELMs beyond learned curves

**Fig 7:**
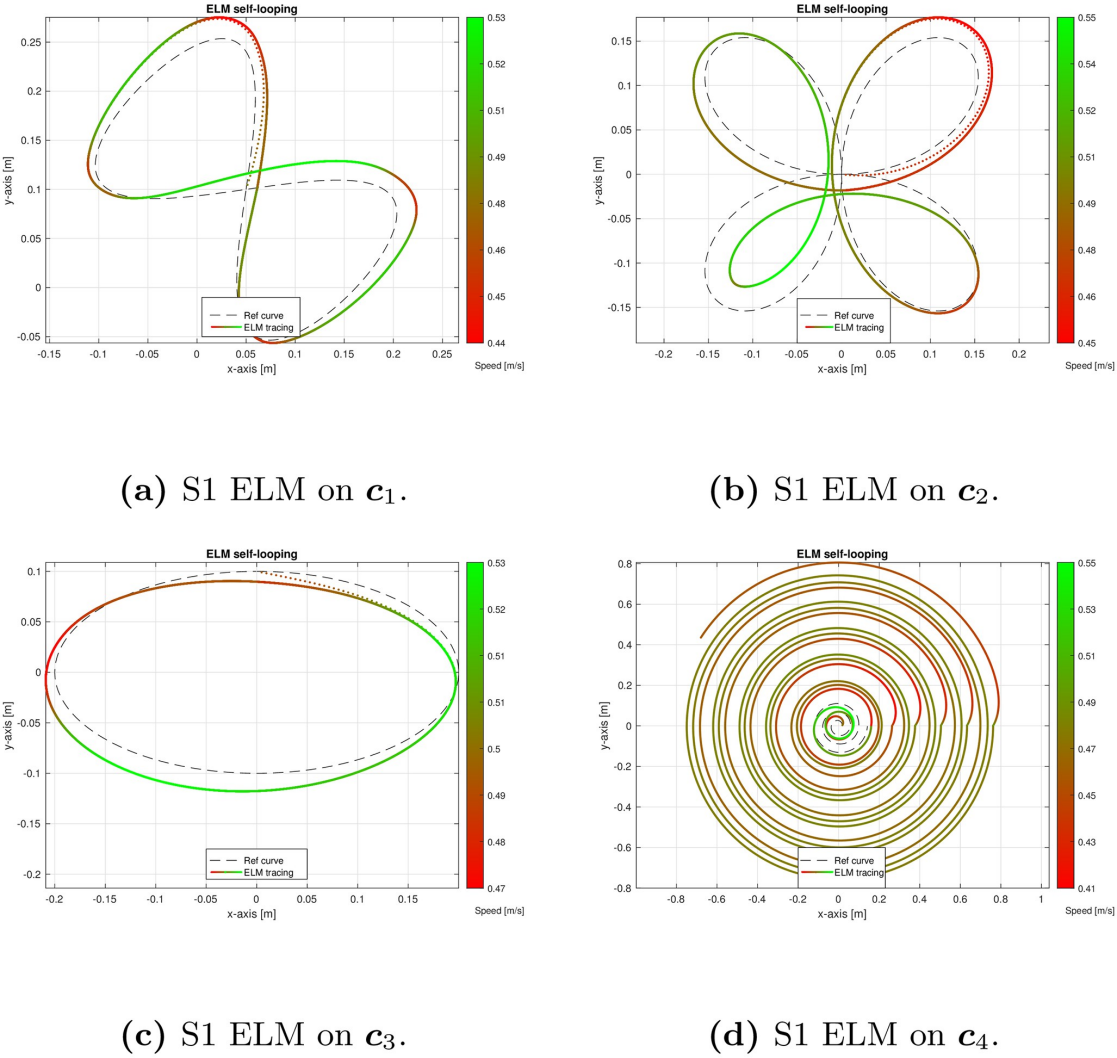
Tracing performance of ELM trained using demonstration data from S1 on (**a**) Affine transformed figure of eight, (**b**) Affine transformed cloverleaves, (**c**) Ellipse and (**d**) Spiral.

To further test the generalization capabilities of this framework, the ELM was tasked to trace out curves not present in the training data using the self-looping method presented in the next subsection and Fig 7. The figures that were chosen are (i) an affine-transformed figure of eight ***c***_1_, (ii) quadrifolium ***c***_2_, (iii) ellipse ***c***_3_ and (iv) a spiral ***c***_4_, to test the ELM’s capability of generalising to new curves with a change in the shape but not the curvature range:

$$\begin{array}{c} \begin{array}{cc} c_{1}\left(p\right)=[\begin{array}{cc} 2.1 & -0.7\\ 3.5 & 2.1 \end{array}][\begin{array}{c} 0.05\;\text{sin}(2p)\\ 0.1\;\text{sin}(p) \end{array}], & c_{2}\left(p\right)=[\begin{array}{c} 0.4\;\text{sin}(2p)\;\text{cos}(p)\\ 0.7\;\text{sin}(p)\;\text{sin}(2p) \end{array}],\\ c_{3}\left(p\right)=[\begin{array}{c} 0.6\;\text{sin}(p)\\ 0.2\;\text{cos}(p) \end{array}], & c_{4}\left(p\right)=0.05\left(p+\pi /4\right)[\begin{array}{c} \;\text{cos}(5p)\\ \;\text{sin}(5p) \end{array}]. \end{array} \end{array}$$

The range of the curvature on these new curves is < 70*m*^−1^. The preprocessing procedure for the ELM data was the same as in the case of the figure of eight complemented with the additional step of trimming those observations for which the speed values are outside of the 10th and 90th interpercentile range. The power law was estimated using the same estimation technique as for the case of the figure of eight. The tracing performance of the ELM trained using Subject *S*1 data for the four new curves is shown in Fig 7(a)–7(d).

These figures showcase our method’s robustness and generalisation abilities and validate its utility in learning invariant mappings to generalize tracing tasks effectively.

The tracing performance of each self-looping ELM can be characterised by a *synthetic* power law evaluated in the same manner as done for the human tracing data. The next question is whether the *human* power law is comparable to this *synthetic* power law. To answer this question, the parameters that the power law regression yields, i.e., the critical curvature *κ*_*c*_ and critical speed *v*_*c*_ are computed from all the trained self-looping ELMs. The human power law parameters are indicated in red while the corresponding *synthetic* power law parameters are in blue in Fig 6(c).

**Fig 8:**
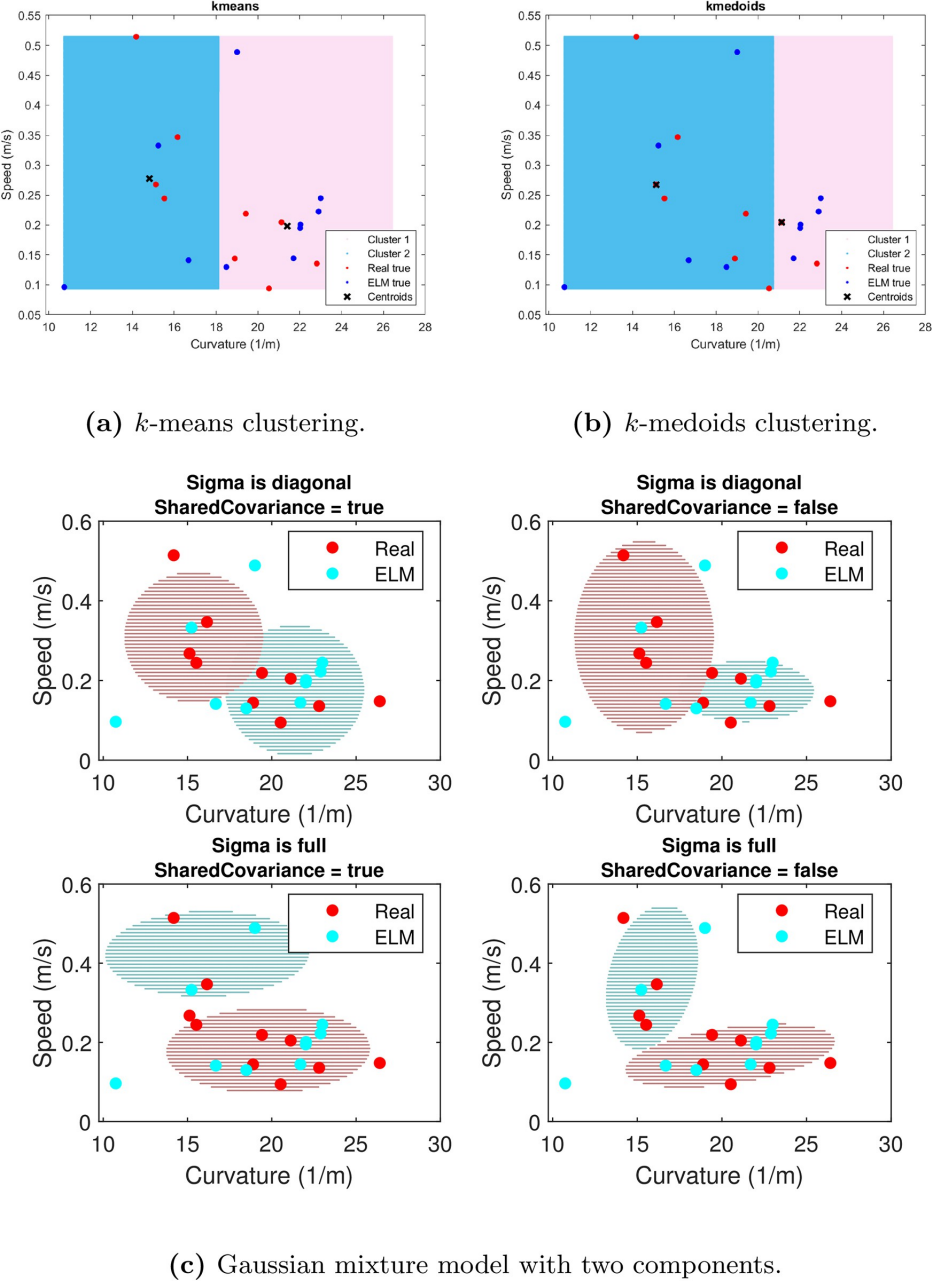
Standard choices of clustering techniques fail to separate the human and ELM-generated power-laws.

The plot of human-synthetic power law in Fig 6(c) motivates an important observation: it is not straightforward to construct some decision boundary that allows the separation of the real and the ELM instances. Furthermore, we use standard clustering techniques to separate the human and ELM-generated curves. As we show in the code available to the reader, none of the standard choices of clustering techniques allows separating between the two types of observations (see Fig 8). In the particular setting of human- and ELM-generated groups of curves, the fact that none of these methods would allow for an accurate separation between them illustrates the indistinguishability between the critical speeds and curvatures of human- and ELM-associated trajectories.

### 4.3 Discussion

In comparison to other methods for learning tracing tasks in the field of Learning from Demonstrations, our contribution is the geometric nature of learning such tracing tasks. Specifically, one of the applications shown in Dynamic Movement Primitives (DMPs) [33] is the learning of the task of tracing a letter of the alphabet from a demonstration. Their main novelty emphasizes the need for a phase variable that allows for scaling in time, i.e., yielding faster or slower executions of the task. The geometric nature of learning in their work is limited to the specification of a start point and a goal along with a scaling term as a difference between the start and goal. The scaling term carries special significance in ensuring invariance properties when generalising. In other words, without the appropriate scaling proportionate to the change in goal position, the DMP distorts the generalised curve into looking very different. Our approach allows greater geometric generalisation in this sense, because we train on invariant geometric properties of the tracing task, i.e., the signed error, the normalised curvature, and the curve parameter.

Independently of the type of machine learning algorithm, feeding a model with numerical values which are not invariant will likely hinder generalisation. To evaluate that, we test our network with a non-invariant. In our work, the invariant formulation is presented in Eqs (8)–(10). We used the standard definition of curvature in Eq (3), which is based on ***c***_*ss*_, i.e., the arc-length *s* rather than the curve parameter *p*. If we simply relied on ***c***_*pp*_, instead of ***c***_*ss*_, the ELM would have failed to generalise even for the same curve, just described at a different speed. To demonstrate this, we trained the ELM on the non-invariant curvature based on ***c***_*pp*_ and tested the curve tracing capability on the figure of eight with different speeds, i.e., by varying *n* in:

$$\begin{array}{c} c_{gen}\left(p\right)=[\begin{array}{c} 0.05\;\text{sin}(2pn)\\ 0.1\;\text{sin}(pn) \end{array}]. \end{array}$$ (22)

**Fig 9:**
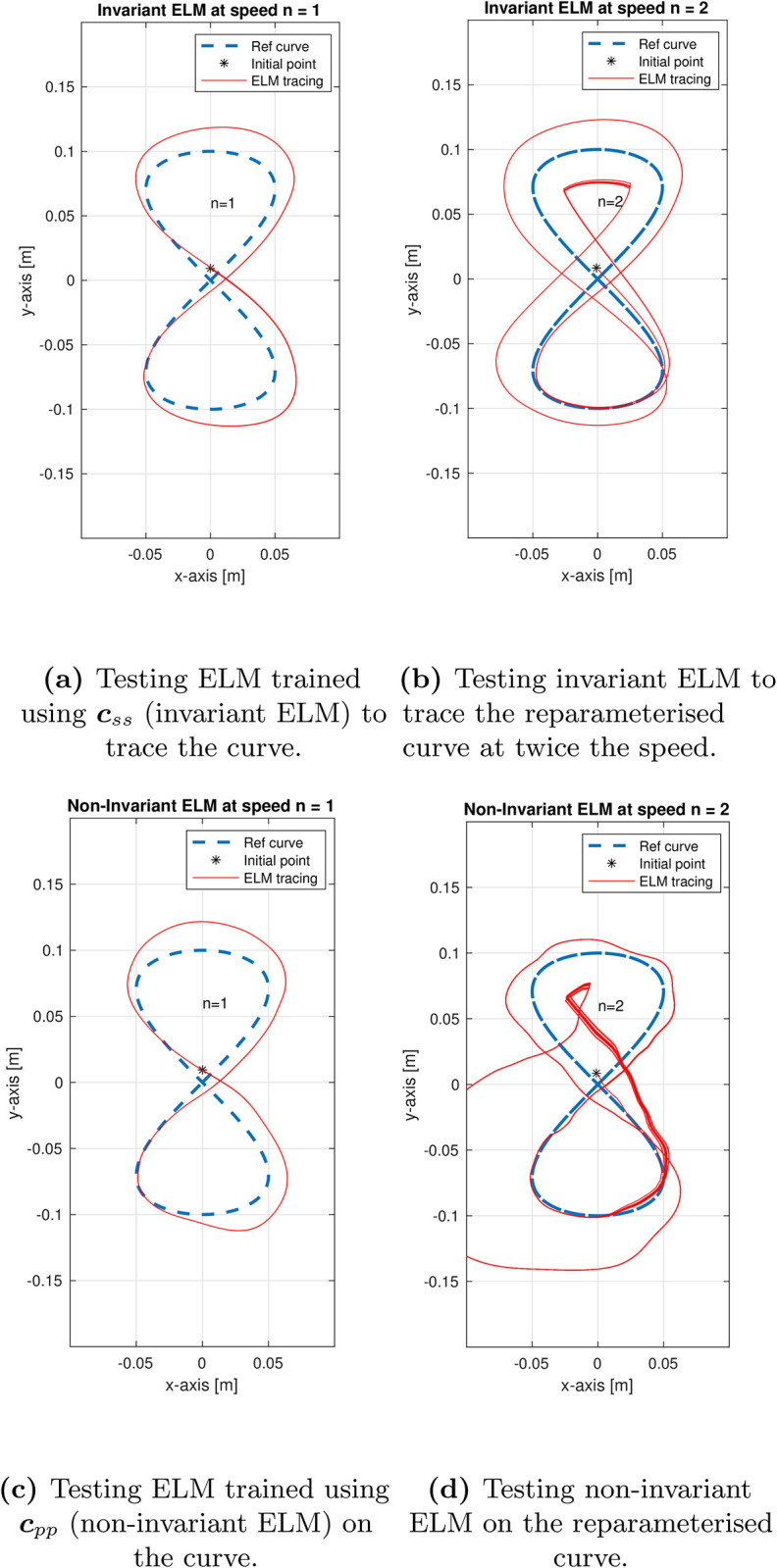
The importance of geometric invariance is demonstrated by the ELM’s failure to generalize even for the same curve, just described with a different speed. In (**a**)-(**b**), the ELM trained using ***c***_*ss*_, successfully traces the figure of eight even at different reparameterisations of *n* = {1, 2} (see Eq 22). However, in the non-invariant ELM (which was trained using ***c***_*pp*_), in (**c**)-(**d**), the generalisation performance declines.

Specifically, for both speeds of *n* = {1, 2}, the invariant ELM (trained using ***c***_*ss*_) successfully traced the figure of eight as seen in Fig 9(a)–9(b). However, the non-invariant ELM (trained using ***c***_*pp*_) had difficulty at a greater speed (Fig 9(d)). We identify this as a future scope to rigorously study the advantages of geometric invariance to tracing generalisation under various reparameterisations of the same or different curve.

## 5 Conclusion

This work contributes to the field of *movement synthesis* for robots by proposing a geometric invariant learning framework to perform planar curve tracing tasks.

We first identify that speed and curvature during a tracing task are related through a 2/3 logarithmic empirical law observed in previous studies in neuroscience. We remark that this law characterises *human-like* curve tracing. Building on this we propose to construct an input-output relation between geometric invariants such as the curvature of the curve at the closest point and the instantaneous speed of the human subject and perform supervised learning on this data. Our tool of choice for learning is an ELM which is a reservoir of neurons constructed so that its training is a single linear regression step of the output layer.

Given a human demonstration of the tracing task on a curve, the ability to generalise to a different curve is important to assert the robustness of the learning paradigm. This is shown by the reservoir’s ability to trace out figures like cloverleaves, ellipses, and a spiral.
